# Supplementary material for: Mechanisms governing the pioneering and redistribution capabilities of the non-classical pioneer PU.1
Source: Nat Commun. 2020 Jan 21;11:402. doi: 10.1038/s41467-019-13960-2 (PMC6972792; doi:10.1038/s41467-019-13960-2)
Supplement: Supplementary file 7 — Source data [file 41467_2019_13960_MOESM7_ESM.zip › Source_Data/FigureS3/FigureS3H_MotifScanOutput/homerResults/motif40.similar.html]

motif40

## Information for motif40

C
T
A
G
G
T
A
C
C
T
G
A
A
T
G
C
C
G
T
A
T
C
A
G
G
C
A
T
G
T
C
A
C
T
A
G
A
C
G
T
G
C
T
A
T
A
G
C
  
Reverse Opposite:  

A
T
C
G
C
G
A
T
T
G
C
A
A
G
T
C
C
A
G
T
C
G
T
A
A
G
T
C
C
G
A
T
A
T
C
G
A
G
C
T
A
C
T
G
A
G
T
C
  

|  |  |
| --- | --- |
| p-value: | 1e-22 |
| log p-value: | -5.253e+01 |
| Information Content per bp: | 1.699 |
| Number of Target Sequences with motif | 53.0 |
| Percentage of Target Sequences with motif | 1.37% |
| Number of Background Sequences with motif | 84.3 |
| Percentage of Background Sequences with motif | 0.19% |
| Average Position of motif in Targets | 248.0 +/- 171.5bp |
| Average Position of motif in Background | 189.5 +/- 162.4bp |
| Strand Bias (log2 ratio + to - strand density) | 0.6 |
| Multiplicity (# of sites on avg that occur together) | 1.06 |
| Motif File: | file (matrix) reverse opposite |

### Similar de novo motifs found

|  |  |  |  |  |  |  |  |
| --- | --- | --- | --- | --- | --- | --- | --- |
| Rank | Match Score | Redundant Motif | P-value | log P-value | % of Targets | % of Background | Motif file |
| 1 | 0.813 | C T A G A G T C C G T A A T G C C G T A A C T G C G A T C G T A G T C A A C G T C G T A A T G C C G T A A T G C | 1e-18 | -42.758524 | 0.47% | 0.00% | motif file (matrix) |
| 2 | 0.879 | G C T A C T A G A G T C G T C A A T G C C G T A C T A G A C G T C G T A C T G A A C G T G C T A A G T C | 1e-13 | -30.061258 | 0.39% | 0.01% | motif file (matrix) |
